# Editorial: Myriad types of cell death in nephropathy and their veiled potential

**DOI:** 10.3389/fendo.2023.1251148

**Published:** 2023-07-21

**Authors:** Jun Wada, Rashmi S. Tupe, Isha Sharma

**Affiliations:** ^1^ Department of Nephrology, Rheumatology, Endocrinology and Metabolism, Okayama University, Faculty of Medicine, Dentistry and Pharmaceutical Sciences, Okayama, Japan; ^2^ Symbiosis School of Biological Sciences (SSBS), Symbiosis International (Deemed University) (SIU), Pune, Maharashtra, India; ^3^ Department of Medicine, Feinberg School of Medicine, Northwestern University, Chicago, IL, United States

**Keywords:** apoptosis, necroptosis, pyroptosis, ferroptosis, regulated cell death, chronic kidney disease, diabetic nephropathy

In the two main forms of cell death, necrosis is characterized by disruption of the plasma membrane, release of intracellular contents, and subsequent immunogenic and inflammatory processes, while apoptosis is a regulated cell death in which dying cells with preserved plasma membrane integrity are rapidly engulfed by phagocytes and orderly cleared without promotion of inflammation. Although necrosis is an accidental cell death, several forms of regulated necrosis, such as necroptosis, pyroptosis, ferroptosis, mitochondrial permeability transition-regulated necrosis (MPT-RN), autosis, and NETosis (neutrophil extracellular traps; NETs), are controlled by specific molecular machinery and programs, which are modulated by their corresponding inhibitor.

Necroptosis is triggered by various stimuli including the activation of death receptors (FAS and TNFRSF1A) and toll-like receptors. Subsequently, the receptor-interacting serine/threonine kinase 3 (RIPK3) is activated, which further activates mixed lineage kinase domain-like pseudokinase (MLKL) and induces membrane rupture.

Pyroptosis is initiated by pathogen-associated molecular pattern molecules (PAMPs) and damage-associated molecular patterns (DAMPs), activation of caspase 1 (CASP1) or CASP11, which cleaves gasdermin D (GSDMD) to produce a 22-kDa C-terminal (GSDMD-C) and a 31-kDa N-terminal fragment (GSDMD-N). Finally, GSDMD-N induces plasma membrane rupture and pyroptosis.

Ferroptosis is induced by iron-accumulation-induced reactive oxygen species (ROS) production and impaired antioxidants system against lipid peroxidation. In the former, lipid oxidation pathway involving acyl-CoA synthetase long-chain family member 4 (ACSL4), lysophosphatidylcholine acyltransferase 3 (LPCAT3), and arachidonate lipoxygenases 15 (ALOX15) is required for lipid oxidation in ferroptosis. In addition, the reduction in protective system including solute carrier family 7 member 11 (SLC7A11), phospholipid hydroperoxidase glutathione peroxidase 4 (GPX4), and transcription factor nuclear factor, erythroid 2 like 2 (NFE2L2, also known as NRF2) is also involved.

In this Research Topic, two review articles focus on diabetic nephropathy. Recently, the concept of “diabetic tubulopathy” has emerged, and “tubulocentric theory” arouses the broad attention, since SGLT2 inhibitors demonstrate the significant therapeutic potential against the progression of diabetic nephropathy. Shen et al. summarize the cellular senescence and regulated cell death (apoptosis, autophagic cell death, necroptosis, pyroptosis, and ferroptosis) in tubular epithelial cells induced by high glucose, lipotoxicity, oxidative stress, and inflammation. Lipid metabolism disorder is a key factor in progression of diabetic nephropathy. Ectopic lipid deposition is aggravated in DN, which further promotes tubule cell inflammation and apoptosis and ultimately aggravates the pathological changes of diabetic nephropathy. Yang et al. sheds light on lipid droplets (LDs) connected to a part of the mitochondria known as the peridroplet mitochondria (PDM). Yang et al. summarize the function of PDM and discuss the possibility of the use of PDM as a therapeutic target.

Zhang et al. discuss about the renal fibrosis, a common feature in the progression of chronic kidney disease (CKD). In recent years, cellular senescence of renal tubular epithelial cells can accelerate the progression of renal fibrosis. To eliminate the senescent cells, calorie restriction and routine exercise, Klotho, senolytics, and senostatics are postulated as interventions. The relation of apoptosis and ferroptosis in the pathogenesis of cellular senescence and renal fibrosis is also discussed.

## Author contributions

JW drafted the editorial. All authors contributed to editing the manuscript. All authors contributed to the article and approved the submitted version.

